# Association between physical activity and body posture: a systematic review and meta-analysis

**DOI:** 10.1186/s12889-023-16617-4

**Published:** 2023-08-30

**Authors:** Mohammad Salsali, Rahman Sheikhhoseini, Parisa Sayyadi, Julie A. Hides, Mahdis Dadfar, Hashem Piri

**Affiliations:** 1 Faculty of Physical Education and Sport Sciences, Allameh Tabataba’i University, Tehran, Iran; 2 Department of Corrective Exercise & Sport Injury, Faculty of Physical Education and Sport Sciences, Allameh Tabataba’i University, Western Azadi Sport Complex Boulevard, Hakim Highway, Tehran, Iran; 3 Department of Health and Sport Medicine, Faculty of Physical Education and Sport Sciences, University of Tehran, Tehran, Iran; 4 School of Health Sciences and Social Work, Griffith University, Queensland, Australia; 5 Menzies Health Institute Queensland, Griffith University, Queensland, Australia; 6 Department of Human Health and Performance, Faculty of Kinesiology, University of Houston, Houston, TX USA

**Keywords:** Physical activity, Body posture, Scoliosis, Lumbar lordosis, Meta-analysis

## Abstract

**Objective:**

To estimate the possible associations between posture and physical activity (PA).

**Design:**

A systematic review and meta-analysis.

**Data sources:**

The search was conducted in seven databases (PubMed, Web of Science, SportDiscus, EMBASE, Scopus, Cochrane Library, and CINAHL) for studies published from inception to January 2023.

**Eligibility criteria for selecting studies:**

Studies were required to meet following criteria: (1) study design: cross-sectional, case control and cohort studies. (2) Participants: people of all ages without any diagnosed diseases. (3) Exposure and outcome: studies that examined the possible effect or correlations between PA, physical inactivity, physical exertion and human body posture.

**Results:**

Sixteen cross-sectional studies, two cohort studies and one case control study involving a total of 16772 participants aged from 6 to 79 years were included. Correlational studies showed that there was a significant relationship between PA and posture (C = 0.100, CI 95% = 0.012–0.186). However, regression studies demonstrated that there was not a significant association between PA and posture (C = 1.00, CI 95% = 0.998–1.002). Three studies investigated the association between PA and the lumbar lordosis and showed that there was not a significant association between the lordosis and PA (CI 95%: -0.253–0.048, *P* = 0.180). In addition, four studies showed that there were not any associations between scoliosis and PA (CI 95%: 0.819, 1.123, *P* = 0.607). The evidence of heterogeneity and publication bias was found among all analyzed data (*P* < 0.05). Also, meta regression was used for age and BMI and the results were not significant.

**Conclusion:**

Although a weak correlation was shown to exist between PA and human posture, the odds ratio indicated that there was not a significant association between PA and human posture. The lack of a significant relationship may indicate that multiple biopsychosocial factors may be involved in human posture. In summary, our study highlights the need for caution when interpreting the results of meta-analyses, particularly when there is significant heterogeneity and publication bias in the included studies.

**Supplementary Information:**

The online version contains supplementary material available at 10.1186/s12889-023-16617-4.

## Introduction

There are significant known health risks associated with a sedentary and inactive lifestyle. The 2020 WHO guidelines stated that some physical activity (PA) is better than none, more PA is better for optimal health outcomes, and recommend reducing sedentary behaviours [1]. In addition to the known health risks, it has been suggested that prolonged sitting can also result in postural changes. It is thought that poor posture is becoming more and more common in society because of changes in human PA brought about using technology in modern lifestyles [2]. It has been proposed that postural changes are the result of changes in muscle length and soft-tissue structures associated with maintenance of static postures, which can lead to muscle imbalances and ultimately to misalignment of the spinal column [3]. It is well accepted that engaging in PA has significant health advantages [4], such as reducing mortality risks [5], improving psychological well-being [6] and mental health [7], lowering blood pressure [8], and improving the survival of cancer patients [9]. For these reasons, increasing PA has been identified as a global health priority [10]. It may seem logical to assume that engaging in PA would also improve posture, yet little is currently known about this important aspect of human health.

Correct body posture plays a vital role in human health. It has been proposed that ideal upright posture is a sign of musculoskeletal health and is one of key indicators of health of the movement system [11]. It is therefore understandable that deviations in human posture could have detrimental effects on health. For example, it has been shown that postural changes associated with aging could lead to an increased risk of falls. An increased risk of falls was observed in those with a decreased lumbar lordosis, increased sagittal vertical axis and increased horizontal distance between the C7 plumb line and the centre of the ankle [12]. It has been proposed that ideal posture is efficient with respect to energy expenditure and recruits the body’s postural muscles [13]. PA is frequently described as skeletal muscle-produced motion of the body that raises energy expenditure. It can be divided into many categories based on its intensity, type, duration, complexity, and purpose [14]. However, whether PA can improve posture may depend on the cause of the postural deviation. Posture could be affected by recruitment and length of muscles, in association with sustained habitual postures, which could be modifiable. In contrast, structural aspects of morphology, such as pelvic inclination angle, age related postural changes and postural deviations associated with structural scoliosis are not likely to be modifiable in response to PA. Additionally, it has been suggested that the presence of postural abnormalities may be associated with some medical conditions, which may also not be modifiable [15]. On the contrary, new evidence suggests that upright postures are considered to be optimal, even though there is not strong evidence linking any specific posture to better health outcomes [16, 17]. Therefore, practitioners should pay greater attention to this issue when designing a plan to address postural disorders.

It is important to also consider that PA could have unintended or adverse effects on human posture. Moreover, it seems that there may be a population-dependent relationship between physical activity (PA) and posture. For example, it has been found that postural misalignment is more likely to develop in athletes, who are physically active. This is thought to be due to athletes frequently adopting postures and recruiting muscles that are distinctive to their sport as part of PA [18]. Athletes from more flexor dominant sports have been found to adopt more kyphotic thoracic postures [19, 20], and it has also been suggested that there could be a relationship between an increased thoracic kyphosis and low back pain [21]. In addition, it has been shown that men with osteoporosis who engaged in high levels of PA in their early and middle adult years were more likely to develop spinal deformities [22]. However, if PA can have a positive effect on human posture, this would be beneficial as clinicians have documented that altered human posture may be associated with low back pain [23], respiratory problems, musculoskeletal pain patterns [2], and pain in the thoraco-cervical-shoulder region [11].

It is not unreasonable to assume that increasing PA could have positive effects on human posture. Generally, PA is also known to be effective in primary and secondary prevention of several chronic diseases [24]. It is recommended that people with osteoarthritis of the lower extremities undertake PA in sufficient amounts [25]. Moreover, it seems that engaging in PA can lead to better muscular function and subsequently may be associated with good posture. For example, postural trunk muscles are activated by being upright and during gait [26] – including paraspinal muscles (erector spinae and multifidus [27, 28]) and the abdominal muscles (transversus abdominis, internal and external oblique [28, 29]). It is therefore possible that PA could affect posture through improved recruitment of postural muscles. Postural muscles such as the multifidus control the segmental motion of the lumbar spine and the lordosis [30] and are negatively affected in conditions like scoliosis [31], low back pain and prolonged bedrest (muscle atrophy and fatty infiltration) [32, 33]. It has been previously proposed that that walking could be used as a form of rehabilitation to improve lumbar paravertebral muscle health [27]. It has also been suggested that prescribing PA could correct some postural abnormalities including spinal scoliosis [34], forward head posture [35], increased lumbar lordosis [36], or knee deformities [37].

In summary, the association between PA and posture is controversial. Ideally, if a positive relationship was shown to exist between PA and posture, this could possibly explain how PA could help to prevent musculoskeletal conditions such as low back pain [38] and possibly falls in the elderly [39]. These conditions place an enormous burden on society. Moreover, recent studies suggest that the ideal posture may differ among individuals. On the other hand, previous studies have consistently highlighted the connection between physical activity and body posture. Therefore, to mitigate the complications and costs associated with postural disorders, it is crucial to evaluate the relationship between posture and various health factors, including the level of physical activity. To the best of our knowledge, no systematic reviews have evaluated the association between PA and posture. Thus, we conducted a review with meta-analysis of existing studies assessing the association between PA and human posture.

## Methods

The Preferred Reporting Items for Systematic Reviews and Meta-Analyses (PRISMA) guidelines were used for this study [40]. The PRISMA statement is intended to provide guidelines for systematic review reporting quality [41]. The review protocol has also a prospective registered in the PROSPERO database (CRD42022329395).

### Eligibility criteria

The requirements for inclusion in the meta-analysis were: (1) study design: cross-sectional, observational and cohort studies. (2) participants: People of all ages without any diagnosed diseases. (3) Exposure and outcome: studies that examined the possible effect or correlation with PA, physical inactivity, physical exertion on/with human body posture. The studies which were based on the qualitative researches, interventional studies, RCTs were excluded. Also, we did not accept the non-English articles. The studies that did not measured the PA level by a standard tool excluded from the study, too.

### Information sources

Our search was performed from inception to 22 January 2023 in seven databases including PubMed, Web of Science, SportDiscus, EMBASE, Scopus, Cochrane Library, and CINAHL, without taking language into account in the first instance. Three authors (MS, PS and MD) independently screened the list of references of selected studies and consulted with expert (RSh) in the research area.

### Search strategy

The search strategy was based on a combination of PA and body posture alignment related keywords with Boolean operators, Quotation marks, and Truncation to achieve a reliable search strategy. The search strategies for the databases PubMed, Web of Science, SportDiscus, EMBASE, Scopus, Cochrane Library, and CINAHL are shown in Supplementary Table 1. In general, the following terms were included in searches: ("physical activity" OR "physical inactivity" OR "physical exertion") AND (postur* OR Alignment OR Malalignment OR deformity OR abnormality) AND (Correlation OR Association OR Regression OR relation*).

### Selection process

All searched records were imported into EndNote 20 (desktop version). The software was used to remove duplicate articles. Three authors (MS, MD and PS) systematically screened the included studies’ titles and abstracts based on the inclusion criteria and the PRISMA 2020 standard protocol [40]. Following that, reviewers individually read, analysed the texts and decided whether to include or exclude the articles. In case of disagreement, the three authors discussed with a fourth reviewer (RSh) to reach a consensus.The reasons for eliminating articles during the full-text screening were noted.

### Data collection process

By using a standard Excel data extraction sheet, two researchers (MS and PS) separately extracted the data and then compared the results to check for consistency. All disagreements were resolved through conversation between the two reviewers and the subject matter expert (RSh). If pertinent information was not provided in the text, it was extracted from the graphs and figures. The following data were retrieved from included studies based on: (1) study characteristics (e.g., publication year and first author’s name, sample size, country, study design and statistical method); (2) participants’ demographic information (i.e., sex and age); (3) PA measurement (e.g., objective measurements, self-reported); (4) Postural assessment (i.e., DXA, mobile phone application, Adams Test, Flexible ruler etc.) and (5) main outcomes (Table 1).

**Table 1:** Characteristics of included studies

Study	Participant	Geographic region	Sex	PA measurement	Postural measurement	Method	Study design	Main outcome
Juskeliene et al., 1996 [42]	791 children aged 6–7 years	Lithuania	417 boys 374 girls	Researcher-Made Questionnaire	Objectively And using posture grid and plumb line	Logistic regression	Cohort study	Physical activity was found to be associated with trunk asymmetry
Silman et al., 1997 [22]	600 subjects aged 15 years and more	19 European countries	300 men, 300 women	European Vertebral Osteoporosis Study (EVOS) Questionnaire	McCloskey/Kanis method	Multiple logistic regression	Cross sectional study	Regular walking in middle-aged and elderly women is associated with a reduced risk of vertebral deformity. By contrast, heavy levels of physical activity in early and middle adult life are associated with an increased risk in men
Ismail et al., 2000 [43]	6937 men from 50–54 years to 75–79 years	19 countries	Only men	EVOS Questionnaire	McCloskey algorithm	Logistic regression	Cross sectional study	Very heavy activity is associated with single/dual vertebral deformity
Latalski et al., 2013 [44]	380 children aged 14	Poland and Czech Republic	175 girls (46.1%) and 205 boys (53.9%	Self-Designed Auditory Questionnaire	Self-Designed Auditory Questionnaire	Chi-square test	Cross sectional study	There is a relationship between physical activity of the child and the occurrence of postural deformity
Araújo et al., 2014 [45]	489 adults aged 18 years or more	Portugal	311 females, 178 males	European Prospective Investigation into Cancer and Nutrition (EPIC) physical activity Questionnaire	-Radiographic measurements -Individual parameters assessment -Postural patterns	Multinomial logistic regression	Cross sectional study	Total and leisure time physical activity were not clearly associated with sagittal posture. However, subjects in the two highest thirds of total physical activity could be protected from nonneutral postural patterns, even though non significantly
Batistão et al., 2016 [46]	288 students between 6 and 15 years	Brazil	59.4% (*n* = 171) were female *N* = 117 male	Researcher-Made Questionnaire	Qualitative assessment (observation)	Logistic regression	Cross-sectional correlational study	Thoracic hyperkyphosis and lumbar hyperlordosis were not associated with any of the analyzed factors Physical activity was significantly associated with lower lower prevalence of valgus knees
Fernani et al., 2017 [47]	100 college students with an average age of 19.38 ± 1.71 years	Brazil	82 females, 18 males	International Physical Activity Questionnaire-Short Form (IPAQ-SF)	Instrumento de Avaliação Postural (IAP)	Chi-square, Pearson correlation test, Spearman correlation test	Cross sectional study	Postural Alterations Are Presented in Students Who Were Regularly Active
Balko et al., 2017 [48]	50 schoolchildren with 10–11-year-old	Czech Republic	25 boys 25 girls	Researcher-Made Questionnaire	Jaroš and Lomíček test and a Saehan metallic Goniometer	Chi-square test	Cross sectional study	Schoolchildren who were less physically active during the week fell into the poor posture category
Poschl et al., 2019 [49]	100 voluntary students between 14 and 17 years of age (mean age 16.0 ± 0.7 years)	Turkey	*N* = 49 females *N* = 51 males	International Physical Activity Questionnaire-Short Form (IPAQ-SF)	New York Posture Rating Test and symmetrigraph	Spearman Correlation	Cross sectional study	Relationship between physical activity level and posture disorders in adolescents
Sidlauskiene et al., 2019 [50]	532 children, aged from 11 to 14 years	Lithuania	*N* = 288 girls *N* = 244 boys	Youth Physical Activity Questionnaire (YPAQ)	Hoeger visual posture assessment method	The Pearson’s and Spearman’s correlation coefficients	Cross sectional study	The teenagers with low physical activity had poorer posture
Tobias et al., 2019 [51]	3861 between self-reported physical activity at age 11 years and onset of scoliosis by age 15 years	UK	NA	Questionnaire based on The Denver developmental screening test/Measured objectively via Actigraphy	Measure scoliosis by DXA	Logistic regression	Prospective Cohort study	Those children who maintained more moderate/ vigorous physical activity were 30% less likely to have developed scoliosis by age 15
Asadi-Melerdi et al., 2020 [35]	346 sixth grade students selected from 10 elementary schools (11–12 years)	Iran	NA	International Physical Activity Questionnaire-Short Form (IPAQ-SF)	Flexible ruler	Pearson correlation test and linear regression model	Cross sectional study	Physical activity levels were also correlated with the sagittal head angle, and kyphosis
Golalizadeh et al., 2020 [52]	400 female high school students (14 to 18 years old)	Iran	Only female	International Physical Activity Questionnaire-Short Form (IPAQ)	-Scoliometer -Debrunner kyphometer -Flexible ruler - Directly studied for genu varum/valgum -Posture grid for assessment asymmetric shoulder	Logistic regression	Cross sectional study	There was no statistically significant association between scoliosis, kyphosis, asymmetric shoulders, and genu varum disorders and physical activity
De Assis et al., 2021 [53]	156 schoolchildren, with an average age of 13.9 years (Between 12 and 17 years old)	Brazil	86(55.1%) being female and 70 (44.9%) males	International Physical Activity Questionnaire-Short Form (IPAQ-SF)	Adams test	Logistic regression	An observational, retrospective case control study	Low physical activity and irregularly active individuals showed an association with scoliosis in schoolchildren
Koumantakis et al., 2021 [54]	112 healthy young adults with median (IQR) age of 20 years (18.2–22 years)	Greece	66 females 46 males	Researcher-Made Questionnaire	Mobile phone application (iHandy level)	Pearson's bivariate correlations	Cross-sectional correlational study	Neither physical inactivity nor leisure time sport activity was related to lumbopelvic posture in participants of this study
Bertoncello et al., 2021 [55]	840 schoolchildren aged 6–12 years	Brazil	477 females, 363 males	Researcher-Made Questionnaire	Corlett And Bishop Body Map Questionnaire	Chi square test	Cross sectional study	Physical activity is a protection factor for postural alteration
Jandrić and Kragulj, 2021 [56]	212 adolescents with range of 10 to 14 years	Bosnia and Herzegovina	164 girls and 48 boys	Questionnaire [57]	Visual screening (Adams bending's test)	Pearson's test of correlation	Cross sectional study	No significant association of scoliosis with the domain and parameters of physical activity
Scaturro et al., 2021 [58]	428 students aged between 11 and 14 years old	Italy	200 females and 228 males	Self-Administered Questionnaire	Adam’s Test, Bunnel’s inclinometer	Logistic regression	Cross sectional study	Unclear association of Physical activity and Scoliosis
Sarvari et al., 2022 [59]	150 high school students aged 13 to 15 years	Iran	Only men	Physical Activity Questionnaire for Adolescents (PAQ-A)	Image J software, Spinal Mouse Device, Objective	Correlation and Structural Equation Modelling	Descriptive correlational study	Physical activity was significantly correlated to forward head posture, kyphosis and lumbar lordosis

### Risk of bias assessment

Two reviewers (MS and PS) used the Joanna Briggs Institute (JBI) Critical Appraisal tools [29] to assess the possibility of bias, with the particular tool chosen based on the layout of each study included in the review (i.e., cohort, cross-sectional and case–control).

### Data synthesis

The relevant data were extracted from eligible studies (including Odds ratio: OR, Confidence Interval: CI, *P*-values, sample sizes, r value). Then, results of studies were pooled using a random-effects model of meta-analysis and the forest plots of correlation and 95% confidence interval (CI) and estimated odds ratio and 95% CI. Q-test was applied to investigate data heterogeneity, while I^2^ was used to determine percentage of variability due to heterogeneity. In the case of data heterogeneity, meta-regression was performed to identify the potential effect of demographic data (BMI, age) on the meta-analysis results. Funnel plot, standard Log Odds ratio and Fisher’s tests were used to check publication bias. Statistical analyses were performed using comprehensive meta-analysis (CMA) software version 3.0 (Biostat Inc, Englewood, New Jersey).

## Results

### Study selection

**Fig. 1:**
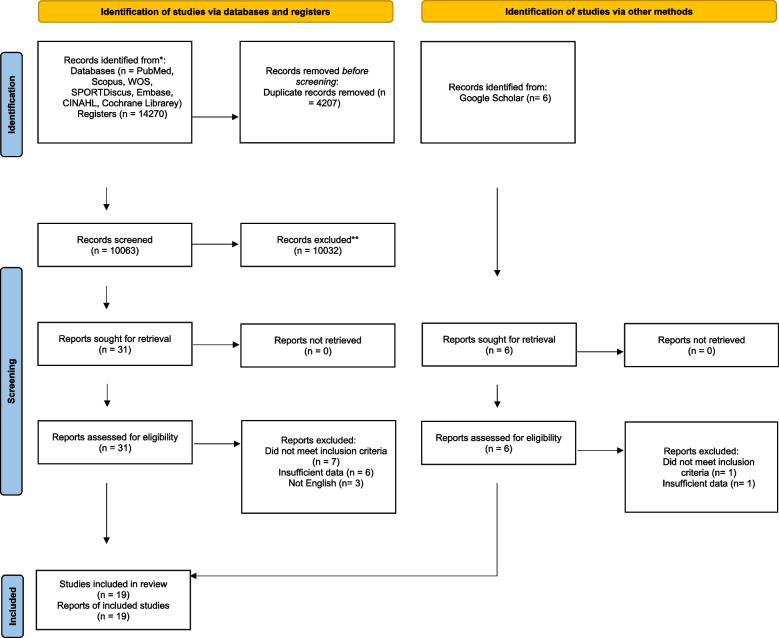
Flow diagram of the included studies

The search strategy identified 14270 studies. After removal of duplicates, 10063 studies remained. Title and abstract screening identified 31 potentially eligible studies. Sixteen of these were excluded due to: not meeting the inclusion criteria (*n* = 7), not having sufficient data (*n* = 6) and studies not written in English (*n* = 3). Fifteen original studies met the inclusion criteria. Six additional studies were included by searching Google Scholar. Two of these were excluded due to: not meeting inclusion criteria (*n* = 1) and not presenting sufficient data (*n* = 1). As a result, in total, 19 studies were included in the review. Figure 1 depicts the PRISMA flow diagram [40], showing the number of studies excluded at each stage of the systematic review and meta-analysis.

### Study characteristics

Publication dates ranged from 1996 to 2022 (median, 2009), with 84% (16/19) of trials published after 2009. Of the eligible studies, 16 studies were cross-sectional, two were cohort studies and one had a case control design. Of the 19 studies and 16772 participants included in this systematic review, two studies included only male [43, 59], one study included only female [52] and 14 studies included both male and female [22, 42, 44–50, 53–56, 58] participants. Two studies did not report on the participants’ sex [35, 51]. Age of the participants ranged from 6 and 79 years. Selected studies were conducted in Bosnia and Herzegovina [56], Brazil [46, 48, 50, 54], Czech Republic [44, 48], Greece [54], Iran [35, 52, 59], Italy [58], Lithuania [42, 50], Poland [44], Portugal [45], Turkey [49], and UK [51]. Two studies involved multi-country collaborations and were conducted across 19 European countries [22, 43]. Nine studies conducted regression analyses [22, 42, 43, 45, 46, 51–53, 58] and ten studies used correlational analyses [35, 44, 47–50, 54–56, 59]. PA was self-reported in all studies and posture was measured in various ways including Dual-energy X-ray Absorptiometry (DXA) [51], Adam’s test [53, 56, 58], questionnaire [44, 55] and qualitative assessment [46] (Table 1).

### Risk of bias

The specific JBI tool was applied according to study design (i.e., cross sectional (*n* = 16), cohort (*n* = 2), and case control (*n* = 1)). In the 75% of cross-sectional studies, exposure and outcome was measured in a valid and reliable way. Also, 68% described the study subjects and the setting in detail. Forty-three percent of these studies did not clearly define the inclusion and exclusion criteria. Although most of the cross-sectional studies (93%) identified confounding factors in their analysis, 43% did not state strategies to deal with these. For the case–control study, confounding factors and strategies to deal with them were not identified. However, this study measured exposure in a standard, valid and reliable way, had an exposure period long enough to be meaningful and used appropriate statistical analyses. Also, exposure was measured in the same way in the cases and controls. Regarding cohort studies, both did not identify confounding factors and did not inform the strategies to deal with them. However, the outcomes were measured in a valid and reliable way. Online supplemental tables contain detailed information on the risk of bias in each study (Supplementary Table 2– 4).

### Description of the selected variables

Overall, five studies evaluated the association between PA and scoliosis. To detect and assess scoliosis the studies used the Scoliometer [52], dual energy X-ray absorptiometry (DXA) [51], Adams Test [53, 56, 58], and the Bunnel inclinometer The lumbar lordosis was assessed using several tools including the flexible ruler [35], spinal mouse [59], radiography [45] and the Bubble inclinometer [54]. Nine studies reported postural deformities using the following measures: McCloskey/Kanis method [22], New York Posture Rating (NYPR) test [49], posture grid and plumb line [42], radiograph measurement [43], Hoeger posture assessment method [50], Instrumento de Avaliação Postural (IAP) [47], The Jaroš and Lomíček test [48] and a questionnaire method [54, 55]. One study had assessed the possible association between PA and valgus knee by observation and qualitative assessment [46]. We reported answers to the JBI tool used to assess the validity and reliability of PA and posture assessment. In JBI, the questions regarding exposure measurement analysed PA assessment, while the questions regarding outcome measurement examined the assessment of posture validity and reliability (Supplementary Tables 2–4).

### Data analysis

Several methods were used to investigate the associations between PA and posture abnormalities. Eleven studies used regression analysis, seven studies used correlational methods for evaluating data and four studies used a Chi-square approach.

Furthermore, three studies that used regression analyses [43, 45, 52] and one study using correlations between variables [35] investigated the associations between PA and several postural alignments (e.g., kyphosis, genuvarum, lumbar lordosis, asymmetric shoulder height, scoliosis) using similar methodologies and populations. Therefore, we were able to perform fixed model meta-analysis for these studies, and this was then added to the main analysis.

### Correlations between PA and body posture

**Fig. 2:**
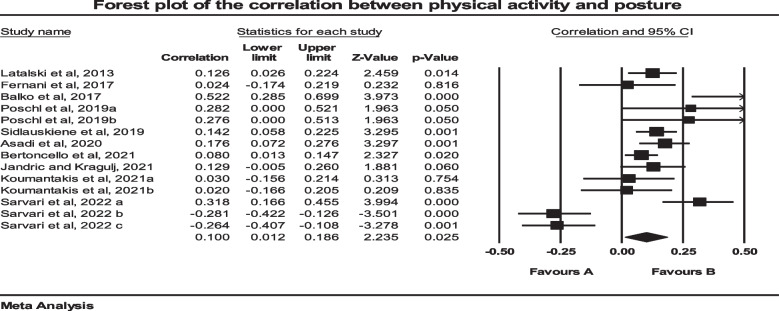
Forest plot of the correlation between Physical Activity and Posture. CI: Confidence interval

**Fig. 3:**
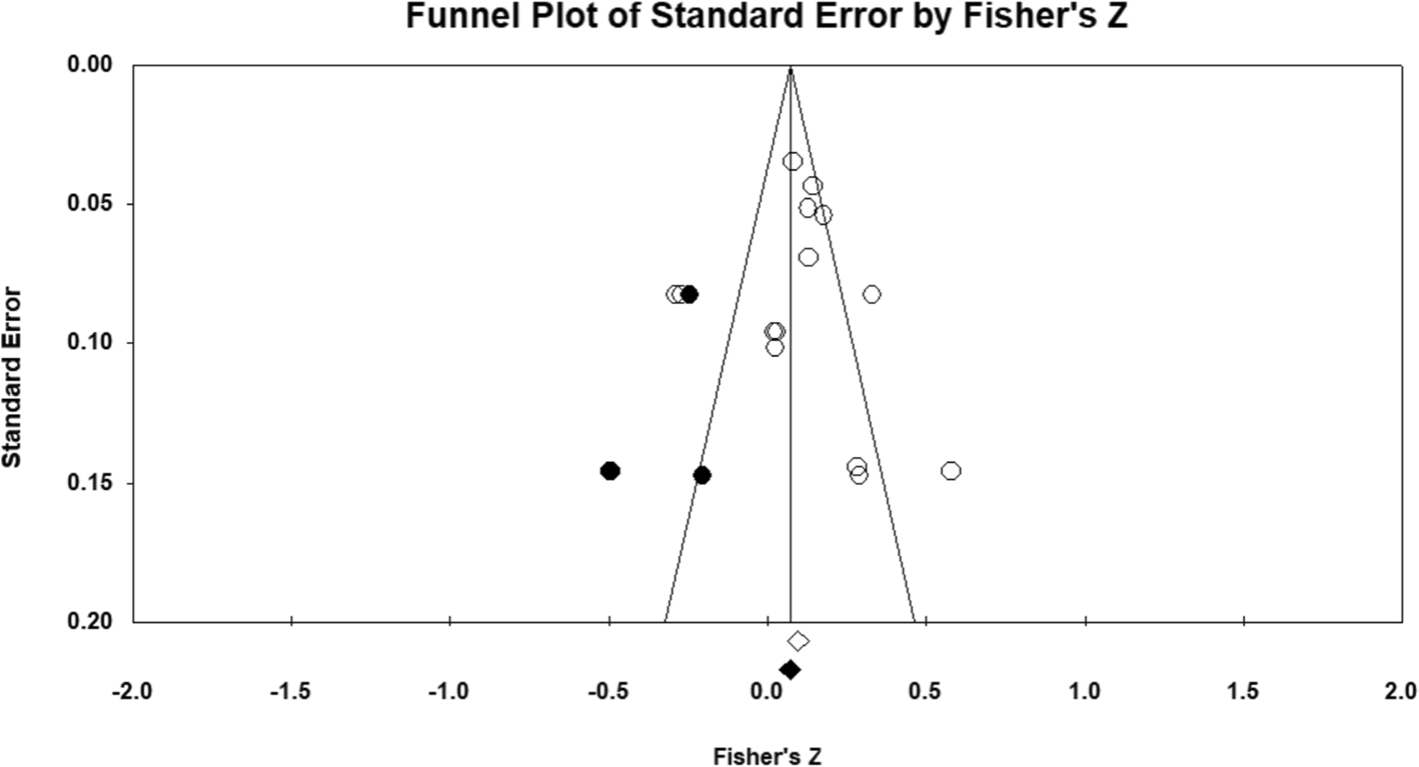
Funnel plot assessing the presence of publication bias in a meta-analysis of correlations between Physical Activity and Posture. The white circles represent the studies that were actually observed, while the gray circles represent the studies that were imputed

Ten studies examined the possible correlations between PA and posture. As two independent groups were included in two studies, both groups were included [49, 54]. Also, three independent groups were included in another study [59]. The total number of participants in these studies was 2822. Prior to conducting the main analysis, we used fixed model meta-analysis to aggregate the data from a study that examined several postural abnormalities separately [35]. We obtained an overall correlation from these data. As seen on the forest plot (Fig. 2), results showed that there were significant correlations between PA and body posture (*p* = 0.025, CI 95% = 0.012–0.186). The majority of these studies reported that there was a positive correlation between PA and good posture [35, 44, 48–50, 55, 59]. However, in some cases PA has negative correlation with the postural deformity [35, 47, 54, 56, 59]. The results revealed that the heterogeneity was significant after taking it into account (*P* = 0.001), (I^2^ = 81.37). However, the main finding was unchanged, suggesting that publication bias may not have had an obvious impact on the results of the meta-analysis (*P* = 0.86) (Fig. 3). Meta regression was used for the variables of age (*P* = 0.057) and BMI (*P* = 0.22). The results showed that there was not a significant effect of these variables on the results.

### regression analyses of pa and body posture

**Fig. 4:**
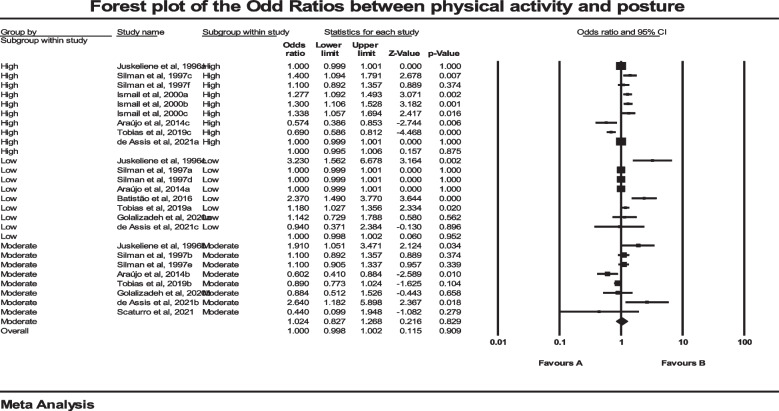
Forest plot regarding the association Physical activity and Posture. CI: Confidence interval

**Fig. 5:**
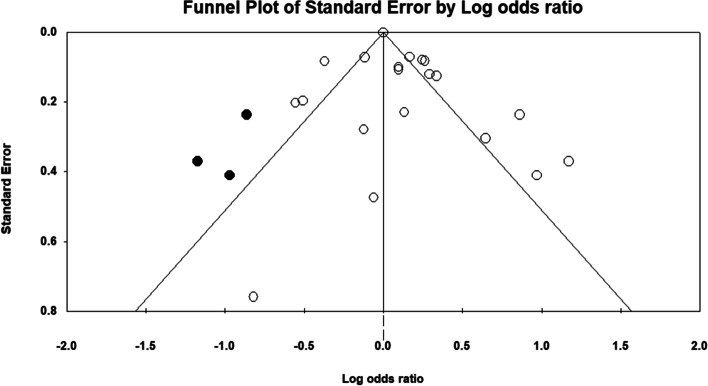
Funnel plot assessing the presence of publication bias in meta-analysis for association of Physical Activity and Posture. The white circles represent the studies that were actually observed, while the gray circles represent the studies that were imputed

Nine studies used regression analyses and odds ratios (OR) to examine the possible associations between PA and posture. The total number of participants in these studies was 13,950. Prior to the main analysis, we used a fixed model meta-analysis to aggregate the data from three studies that had investigated several postural abnormalities separately in same population [43, 45, 52]. Eventually, the pooled estimated OR was 1 (*P* = 0.909, CI 95% = 0.998–1.002). Analysis of the data from these studies with the help of a forest plot showed that there was not a significant association between PA and posture (Fig. 4). After examining the heterogeneity, the results showed that the heterogeneity was significant (*P* = 0.001), (I^2^ = 78.66). Meta regression was conducted for age, and the results demonstrated that participant age did not have a significant effect on the obtained results (*P* = 0.95). In addition, we examined PA at three levels as subgroups, and there was not a difference between groups with low, medium and high intensities of PA. Likewise, a funnel plot and Standard Log of OR showed that there was not a publication bias (*P* = 0.11) among the results of the eligible studies (Fig. 5).

### Association between PA and scoliosis

**Fig. 6:**
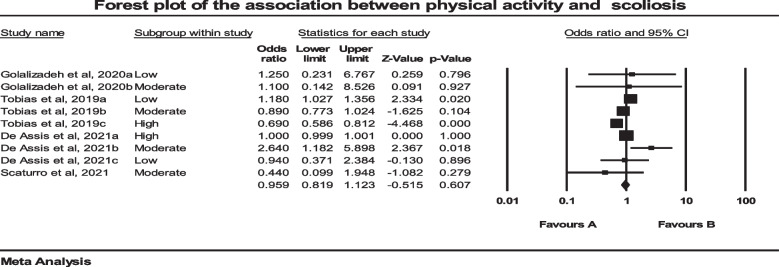
Forest plot regarding the association of Physical Activity and Scoliosis. CI: Confidence interval

**Fig. 7:**
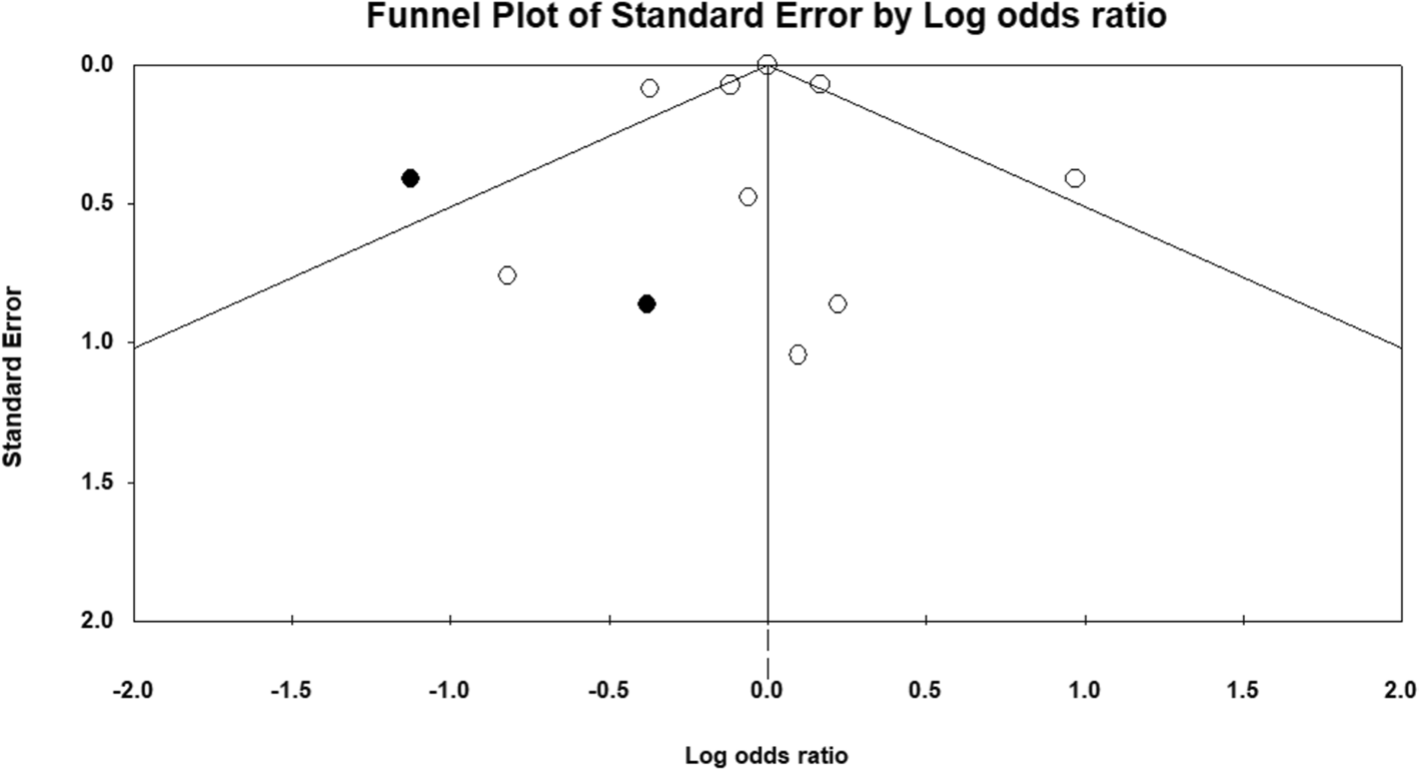
Funnel plot assessing the presence of publication bias in meta-analysis for Physical Activity and Scoliosis. The white circles represent the studies that were actually observed, while the gray circles represent the studies that were imputed

Four studies investigated the association between scoliosis and PA [51–53, 58]. In two studies, three independent groups were included [51, 53]. Also, two independent groups were included in one study [52]. The total number of participants in these studies was 4845. The pooled estimated OR was 0.959 (*P* = 0.607, CI 95% = -0.819–1.123). Analysis of these data using a forest plot showed that there was not a significant association between scoliosis and PA (Fig. 6). Furthermore, significant evidence of heterogeneity was found among the data (*P* = 0.001), (I^2^ = 77.090) from the eligible studies. An asymmetrical funnel plot and insignificant value of the Standard Log of Odds ratio (*P* = 0.91) showed that there was not a clear sign of publication bias (Fig. 7).

### Association between PA and lumbar lordosis

**Fig. 8:**
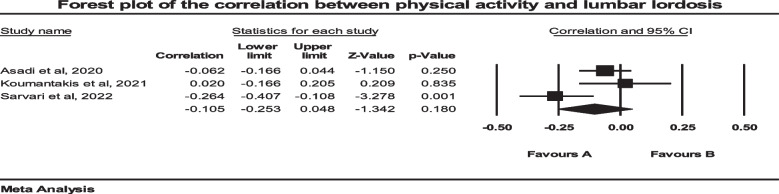
Forrest plot regarding the correlation of Physical Activity and Lumbar Lordosis. CI: Confidence interval

**Fig. 9:**
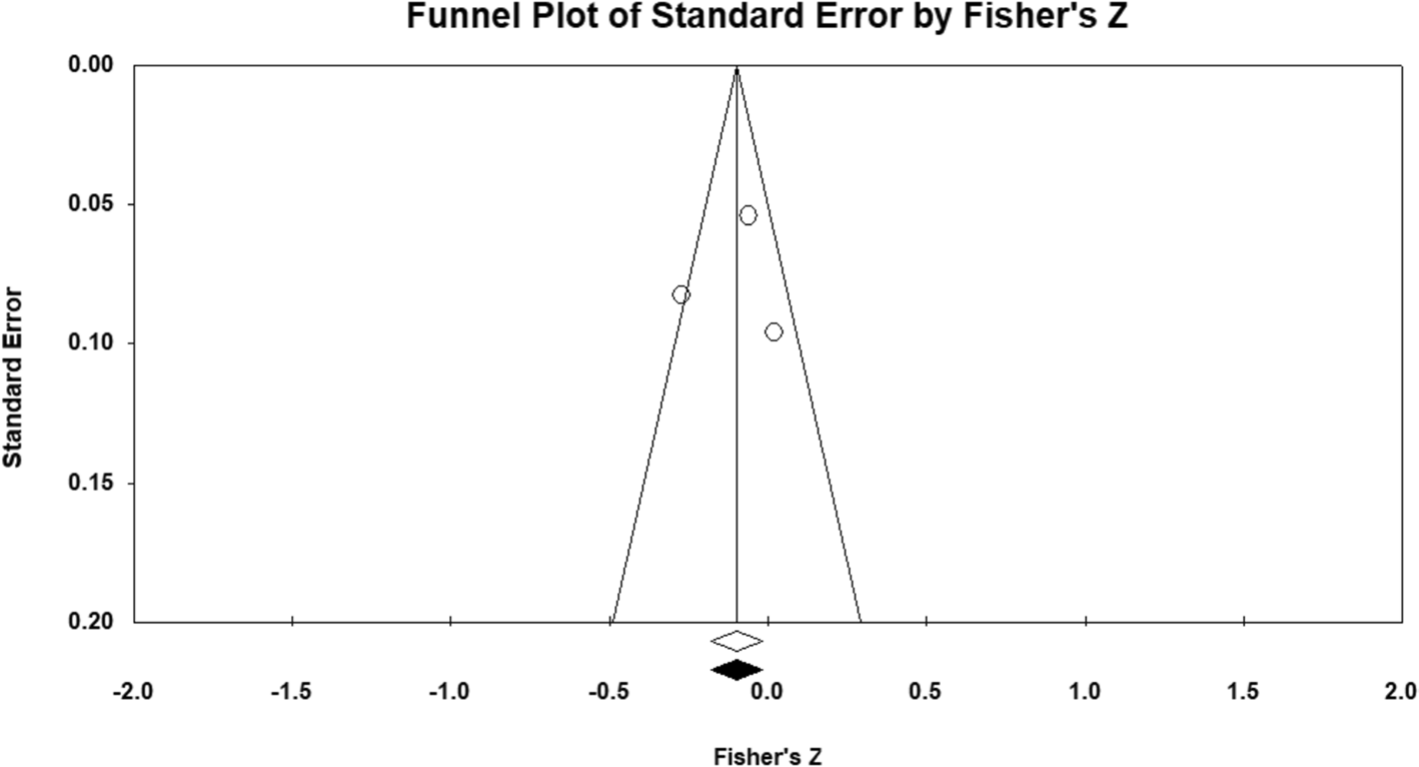
Funnel plot assessing the presence of publication bias in meta-analysis for Physical Activity and Lumbar Lordosis

Three studies investigated the association between the lumbar lordosis and PA [35, 54, 59]. The results of data synthesis of 608 participants (Fig. 8) showed that engaging in PA may not have a significant relationship with the lumbar lordosis (*P* = 0.180, CI 95% = -0.233–0.043). Furthermore, evidence of heterogeneity was found among the data (*P* = 0.042), (I^2^ = 68.40). An asymmetrical funnel plot indicated possible publication bias (Fig. 9), but the value of Fisher's test of the intercept was not significant (*P* = 0.89).

## Discussion

This study pooled the results of studies that examined the possible correlations/associations between PA level and postural alignment in human beings. While the methodological quality of the quantitative studies included in the current systematic review and meta-analysis was deemed to be sufficient, there was wide variation evident in the studies in terms of the intensity of PA undertaken, the types of postural alignment assessed, techniques used to assess posture, and the age range of the participants. The results of the meta-analyses showed that there were significant but weak correlations present, but no other significant associations were found between the level of PA and postural alignment in the study populations. Moreover, this study did not find a significant correlation between PA level and the presence of scoliosis.

The current investigation is the first meta-analysis to examine the association between PA and postural alignment. According to our results, ten studies showed that PA had significant but weak correlations with postural deformity (*p* = 0.025, CI 95% = 0.012–0.186). These studies suggest that PA may be correlated positively with changes in body posture in people of different ages engaged in a variety of activities. Results suggested that a lack of PA may be one of the possible causes of development of postural deviations [44]. For example, students who participated in sports only once a week or less tended to have poor posture [48]. Similarly, previous studies reported that there was a connection between PA and the development of postural deviations [35, 44, 49, 54, 59]. PA levels and the sagittal head angle were found to be negatively correlated, suggesting that as PA increased, the sagittal head angle decreased. The findings of this study also indicated a positive link between the increased thoracic kyphosis angle and increased levels of PA [35]. Based on observations such as these, it has been proposed that PA serves as a protective factor against postural spinal deviations. For example, the results of a study of 150 high school boys demonstrated that increasing PA can lessen the magnitude of increased angles of lumbar lordosis and thoracic kyphosis [59]. Of interest, whereas PA was shown to be associated with increased angles of thoracic kyphosis and a forward head posture, there was not a clear correlation with the size of the lumbar lordosis [59]. Taken together, it seems that although correlations between PA and human body posture have been observed, these can be considered as weak at best.

Apart from the proposed positive effects of PA on posture, it is also possible that undertaking some forms of PA could be associated with inducing postural deformities. Over-recruitment of muscles that traverse the lumbar spine, such as the oblique abdominal muscles, can induce postural changes such as flexion of the thoracolumbar junction or posterior tilt of the pelvis. Activities that strengthen the pectoral muscles without also strengthening the opposing thoracic extensor muscles could lead to muscular imbalance and hyperkyphosis of the thoracic spine [35]. This could be exacerbated by activities such as sitting working on a computer for extended periods of time. In support of this, physical inactivity was linked to increases in posterior pelvic tilt, according to a study of older industrial employees with flexion-related low back pain [60]. In contrast, a cross-sectional study of 112 healthy young individuals between the ages of 18 and 22 revealed no association between lumbopelvic position and recreational sport participation [54]. It is possible that postural deviations associated with muscle imbalances, recruitment and altered muscle length would be more amenable to changes using exercise interventions than postural deviations associated with bony or structural changes [35, 54].

Two additional factors that may influence the effects of PA on study participants is their age and sex. Interestingly, one study reported that while boys had higher PA levels than girls [44], school aged boys were more likely to have poor posture. A cross-sectional study including 400 female students between the ages of 14 and 18 found that PA was not substantially associated with asymmetric shoulder height, hyperlordosis, genu varum, or genu valgum [52]. Additionally, a cross-sectional study examined 288 students with the mean age of 10.6 years among both boys and girls. It revealed that PA was unrelated to hyperkyphosis, forward head posture, or iliac crest elevation. However, results showed that there more valgus deformity in those who were active [46]. Soccer is the most popular sport among physically active people, so it can be hypothesized that playing this game, which mostly involves striking the ball with the medial aspect of the foot, may help to lessen knee valgus stress [46, 61]. Also, in 489 Portuguese adults (311 females, 178 males), PA and sagittal spinopelvic alignment were examined. Results indicated that the sagittal vertical axis was higher in the low PA group or in those who reported spending more time sitting [45]. Increasing age is associated with the likelihood of developing scoliosis in adolescents [56] and a correlation between the size of the lumbar lordosis and body weight and BMI has previously been reported in people still growing [62]. Therefore, we conducted a meta regression for to assess the correlation between human body posture and BMI (*P* = 0.22) and age (*P* = 0.057), however, the result was not significant. Furthermore, results of regression studies showed that PA was not associated with postural alignment (*P* = 0.909, CI 95% = 0.998–1.002).

An additional factor, which can have a variety of effects on postural deviations, is the intensity of PA undertaken. A comprehensive study conducted in 36 centers from 19 European countries revealed that men who engaged in very high levels of PA during their early and middle adult years had an increased risk of developing spinal deformities [22, 43]. Conversely, women who engaged in high levels of physical exercise did not experience an increase [22], suggesting that there is a complex link between PA and spinal deformity that is sex and intensity dependent. However, there was little evidence linking overall leisure-time PA with sagittal posture. Results from one study did indicate that higher levels of overall PA could possibly offer some protection against developing postural abnormalities [45]. When PA was divided into three subgroups (low, medium, and high intensity), there was no evident difference between the groups. It has been proposed that age may affect posture [45]. However, in the current investigation, a separate meta regression examining the association between age and posture using data from regression studies failed to show a statistically significant result (*P* = 0.95).

Finally, the association between scoliosis and PA is controversial. An observational case control study determined that students with low PA levels were more prone to developing scoliosis. In this study, the group with scoliosis contained the highest proportion of students who did not participate in regular PA [53]. However, sedentary individuals did not exhibit this connection [63]. It has also been reported that presence of scoliosis was unrelated to the types and amounts of PA [56]. A cross-sectional study conducted in Italian schools also found that the association between PA and scoliosis was unclear [58]. In the current investigation, we conducted further meta-analyses for PA and scoliosis, but the results showed that there was not a significant association between these variables (*P* = 0.607, CI 95% = -0.819–1.123). Moreover, few studies have investigated the possible association between the size of the lumbar lordosis and PA. The results of his meta analyses indicated that PA was not correlated with the size of the lumbar lordosis (*P* = 0.180, CI 95% = -0.233–0.043).

The statistically significant heterogeneity and publication bias found among the studies in our meta-analysis emphasize the importance of interpreting the results with caution. The presence of heterogeneity implies that there could be variations between the studies that may affect the conclusions drawn from the meta-analysis. These variations could be due to differences in study design, population characteristics, or the methods used to evaluate physical activity and body posture. To address this issue, we utilized a random-effects model and conducted subgroup analyses to investigate potential causes of heterogeneity and identify any patterns in the data. On the other hand, publication bias may indicate that studies with insignificant or negative results were less likely to be published, ultimately resulting in an overestimation of the association between physical activity and body posture. To address publication bias, we employed funnel plots and Egger's test, revealing that there may be some degree of publication bias in the literature. As a result, we interpreted the results of our meta-analysis cautiously and suggested that further research be conducted to confirm our findings. Despite these limitations, our meta-analysis offers valuable insights into the link between physical activity and body posture. Our findings suggest that physical activity may have no positive impact on improving body posture, which could have significant implications for the prevention and management of postural disorders. However, additional research is necessary to validate our findings and investigate potential moderators of the relationship between physical activity and body posture.

### Strengths and limitations

A strength of the current investigation is that it included large representative sample sizes from more than 20 different countries. Additionally, this is the first comprehensive review with meta-analysis to our knowledge to summarize the association between PA and posture in people aged from 6 to 79 years. We conducted a meta-analysis and compiled the pooled association of PA with postural deviations in the included outcomes, despite the fact that some outcomes were not consistently assessed across the studies. We adhered to a protocol that was registered on PROSPERO and carried out this systematic review in accordance with the PRISMA recommendations. We conducted thorough searches across seven reliable databases.

There are some limitations of our study. Firstly, the current study only evaluated original publications in English-language peer-reviewed journals and excluded other scientific literature such as books, conference works, and textbook chapters. Future research could consider a wider range of academic sources and inclusion of other languages. Additionally, the majority of the studies used self-reported questionnaires to estimate the amount of PA of participants. It is therefore possible that recall bias and preference bias may have affected the results Also, we excluded the articles where participants had diagnosed diseases. It is possible that subgroups of more homogenous participants may show different results. For example, it has been reported that posture is often affected in people with Parkinson’s disease, with the trunk tipped anteriorly due to greater pelvic flexion. In those diagnosed with the condition, the amount of anterior tilting of the head and trunk was proportional to the severity of depression [60], highlighting the importance that mental health plays in the posture of human beings [51, 57]. Another point to consider is that none of the studies included in this meta-analysis measured physical activity levels using quantitative tools, and the validity of measurement tools may impact the results of these studies. Another potential limitation of this study is that the generalization or omission of different body postures may not be methodologically sound. When eligible studies investigating different body postures are combined, the potential effects of these differences should be explored using subgroup analyses or sensitivity analyses. However, this was not possible in our study due to the lack of available data. Finally, when generalizing the results of our study, it is important to consider that out of the 19 studies included in the analysis, 17 studies were conducted on individuals under the age of 18. As such, further research is necessary, especially in adults or in comparing the relationship between physical activity and posture in different age groups, to fully investigate this topic.

## Conclusion

The results of the meta-analyses showed that there were significant but weak correlations, but not significant associations between levels of PA and postural alignment in the included study populations. A possible explanation for these findings is that multiple biopsychosocial factors may impact human body posture. Although there are many known benefits of PA, the effects of PA alone on posture were not strong in isolation. Future studies investigating variables affecting posture could include more comprehensive biological (e.g., specific medical conditions), psychological and social factors in order to understand these complex relationships. In summary, our study highlights the need for caution when interpreting the results of meta-analyses, particularly when there is significant heterogeneity and publication bias in the included studies.

### Supplementary Information

**Additional file 1: Supplementary Table 1.** The raw data used for examining relationship between physical activity and posture. **Supplementary Table 2.** The raw data used for examining Odd ratios between physical activity and posture. **Supplementary Table 3.** The raw data used for examining corelation between physical activity and spinal scoliosis. **Supplementary Table 4.** The raw data used for examining corelation between physical activity and lumbar lordosis.

## Data Availability

The datasets generated and analysed during the current study are available in the supplementary file 1.
